# The Role of Serum Th1, Th2, and Th17 Cytokines in Patients with Alopecia Areata: Clinical Implications

**DOI:** 10.3390/cells10123397

**Published:** 2021-12-02

**Authors:** Anna Waśkiel-Burnat, Marta Osińska, Anna Salińska, Leszek Blicharz, Mohamad Goldust, Małgorzata Olszewska, Lidia Rudnicka

**Affiliations:** 1 Department of Dermatology, Medical University of Warsaw, Koszykowa 82A, 02-008 Warsaw, Poland; anna.waskiel@wum.edu.pl (A.W.-B.); marta.osinska1@gmail.com (M.O.); salinska.anna@gmail.com (A.S.); leszek.blicharz@wum.edu.pl (L.B.); malgorzata.olszewska@wum.edu.pl (M.O.); 2 Department of Dermatology, University Medical Center of the Johannes Gutenberg University, 55122 Mainz, Germany; mohamad.goldust@usb.ch

**Keywords:** cytokine, chemokine, immunology, interleukin

## Abstract

Alopecia areata is a type of non-scarring hair loss. The dysregulation of numerous systemic Th1 (IL-2, IFN-γ, TNF, IL-12, and IL-18), Th2 (IL-4, IL-5, IL-6, IL-9, IL-10, IL-13, IL-17E, IL-31 and IL-33) and Th17 (IL-17, IL-17F, IL-21, IL-22, IL-23 and TGF-β) cytokines was observed in patients with alopecia areata. Positive correlations between the severity of alopecia areata and an increased serum level of various cytokines including IL-2, TNF, IL-12, IL-17, and IL-17E were reported in the literature. An increased serum level of numerous cytokines, such as IL-2, IL-6, TNF, IL-12, IL-17E, and IL-22, was described as positively correlated with the duration of the disease. Moreover, it was shown that increased pre-treatment serum level of IL-12 was a positive, while increased serum levels of IL-4 and IL-13 were negative prognostic markers for the efficacy of diphenylcyclopropenone. In conclusion, alopecia areata is associated with the dysregulation of systemic Th1, Th2 and Th17 cytokines with their role in the pathogenesis, clinical manifestations and prognosis of the disease. Available data indicate the most significant role of serum IL-2, TNF, IL-12, IL-17, and IL-17E as markers of disease activity. The serum levels IL-4, IL-12 and IL-13 may be useful as potential predictors of diphenylcyclopropenone efficacy.

## 1. Introduction

Alopecia areata is a type of non-scarring hair loss that affects any hair-bearing area [1]. The prevalence of the disease varies between 1% and 2% in the general population [2,3], with alopecia areata being one of the most common forms of hair loss, diagnosed in 18.2% of cases [4]. Based on the extent of hair loss, the disease is classified into: patchy alopecia areata (with partial scalp hair loss), alopecia areata totalis (with complete scalp hair loss), and alopecia areata universalis (with complete scalp and body hair loss) [1]. Various patterns of hair loss may be distinguished: patchy, ophiasis (band-like hair loss in the parieto-temporo-occipital area), ophiasis inversus-sisaipho (band-like hair loss in the fronto-parieto-temporal area), reticulate, and diffuse [1]. The course of alopecia areata is difficult to predict. The most important factors associated with a poor prognosis include the extent of hair loss (extensive alopecia areata, alopecia totalis, or alopecia universalis), long duration of the disease, and young age at the first onset [5]. Numerous topical, intralesional and systemic agents are currently used in alopecia areata treatment. Topical therapy includes corticosteroids, minoxidil and immunotherapy [6]. Contact immunotherapy with diphenylcyclopropenone (DPCP) and squaric acid dibutylester (SADBE) is mainly recommended for limited hair loss [7]. By some authors, it is considered as first-line therapy in children with alopecia areata. In systemic therapy of alopecia areata, corticosteroid and non-corticosteroid immunosuppressive agents such as methotrexate, cyclosporine and azathioprine are most commonly recommended [7]. Recently, the efficacy of numerous cytokine-targeted medications, such as Janus kinase (JAK) inhibitors, has been described in patients with alopecia areata [8].

Alopecia areata is characterized by the presence of perifollicular and intrafollicular infiltrates, mainly consisting of CD4+ T helper 1 (Th1) cells and CD8+ cytotoxic T cells, respectively [9]. Moreover, Langerhans cells, macrophages, eosinophils and natural killer cells may be detected [10].

The precise etiopathogenesis of alopecia areata is not fully described [11]. The disease is considered as a T-cell-mediated, autoimmune condition with a genetic predisposition and an environmental trigger [1,9]. In healthy individuals, the anagen hair follicle is considered as a site of relative immune privilege with a limited number of antigen-presenting cells as well as locally generated immunosuppressants (e.g., alpha-melanocyte-stimulating hormone, transforming growth factor beta 1, and insulin-like growth factor 1), which induce a very low expression of major histocompatibility complex (MHC) classes I and II molecules [9,12,13,14]. In alopecia areata, in the case of the insufficient activity of immunosuppressive molecules, proinflammatory cytokines including substance P and Interferon-gamma (IFN-γ) induce the ectopic expression of MHC class antigens and the over-expression of adhesion molecules in hair follicle keratinocytes and dermal papilla cells [13]. It results in activation of the cytotoxic, CD8+ T cell pathway and increased migration of antigen presenting cells to the area. The antigen presenting cells process autoantigen from the follicle and subsequently present and activate the naive T cell to differentiate into Th1, T helper 2 (Th2), T helper 17 (Th17), or regulatory T cells [13].

It was suggested that Th1 cells have an essential role in the development of alopecia areata. They produce IL-2, IFN-γ, IL-12, IL-18, and IL-23, which positively feed back to promote further Th1 cell differentiation. Moreover, Th1 cytokines stimulate the production of other proinflammatory cytokines (e.g., TNF and IL-1 alpha and beta), which inhibit hair follicle proliferation [13,15]. Proinflammatory cytokines induce condensation and distortion of the dermal papilla, vacuolization of matrix cells with a decrease in matrix size, disruption of follicular melanocytes, and abnormal keratinization of the follicle bulb and inner root sheath [13,16].

More recent studies proposed that Th2 and Th17 cells are also involved in the pathogenesis of the disease [8].

Th17 cells secrete the pro-inflammatory cytokines such as IL-17, IL-22, and IL-23, which play important roles in the development of inflammatory and autoimmune diseases such as alopecia areata [13].

Th2 cells produce IL-5, IL-6, IL-10, and IL-13, which participate in the conversion of B cells to activated, antibody-producing plasma cells. The role of humoral immunity in pathogenesis of alopecia areata has not been fully confirmed [12,13]. However, in recent studies an increased Th2 profile in individuals with alopecia areata was described [17,18,19]. A frequent coexistence of alopecia areata and atopic dermatitis suggests the common pathogenesis of these conditions with the potential role of Th2 cytokines [18]. On the contrary, Th1 cells which play important role in alopecia areata inhibit the effects of ongoing Th2 cell responses [20]. Thus, the role of Th2 cells in alopecia areata remains uncertain.

Alopecia areata is considered as an organ-specific disease limited to the hair follicles [13]. However, recent studies have indicated that the disease is associated with systemic immune activation with the dysregulation of serum cytokine levels [21].

The clinical role of serum Th1, Th2, and Th17 cytokines in patients with alopecia areata is discussed in this review.

## 2. Cytokines in Alopecia Areata

### 2.1. Th1 Cytokines

A summary of current literature considering the serum Th1 cytokine levels in patients with alopecia areata is presented in Table 1.

#### 2.1.1. Interleukin 2 (IL-2)

IL-2, discovered in 1976, is an interleukin produced by activated T cells [22]. It is an important mediator in the growth, development, and activity of T and B lymphocytes, natural killer cells, and lymphokine-activated killer cells [11]. IL-2 mediates antigen-specific T-lymphocyte proliferation and modulates the expression of IFN-γ and major histocompatibility antigens [11].

Numerous studies described an increased serum level of IL-2 in patients with alopecia areata in comparison with healthy controls [11,22,23,24,25,26,27]. Moreover, higher levels of IL-2 mRNA were detected in peripheral blood mononuclear cells in patients with alopecia areata compared to control subjects [24]. In the study of Teraki et al. [23], an elevated serum level of IL-2 was observed only in patients with alopecia universalis in comparison with healthy individuals and patients with localized alopecia areata. Conversely, no significant difference was present in the serum level of IL-2 between patients with localized alopecia areata and healthy controls. In contrast to other studies, Loh et al. [28] showed a decreased serum level of IL-2 in patients with alopecia areata compared to the control group.

A study performed by Gautam et al. [25] revealed a positive correlation between the serum level of IL-2 and the severity of hair loss. Tembhre et al. [22] showed a positive correlation of serum IL-2 level with the total disease duration and the number of hairless patches on the scalp.

Askin et al. [27] reported a decrease in the serum level of IL-2 in patients with alopecia areata after tofacitinib treatment. However, no significant relationship between the change in interleukin level and the change in the Severity of Alopecia Tool (SALT) scores was observed.

IL-15 is structurally similar to IL-2. Both cytokines signal through two shared receptor subunits, the IL-2/15β chain (CD122) and the common γ chain (γC) [29]. In patients with alopecia areata, an increased serum level of IL-15 compared to healthy controls was described [27,30,31,32].

#### 2.1.2. Interferon Gamma (IFN-γ)

IFN-γ is an activator of macrophages and inducer of class II MHC molecule expression. It is produced predominantly by natural killer and natural killer T cells as part of the innate immune response, and by CD4+ Th1 and CD8+ cytotoxic T lymphocyte once antigen-specific immunity develops [31].

According to the majority of previously published studies, an increased serum level of IFN-γ was observed in patients with alopecia areata compared to healthy controls [22,23,25,26,31,32,33,34,35,36,37,38,39,40,41]. Higher IFN-γ expression was also detected in peripheral blood mononuclear cells in patients with alopecia areata in comparison with the control group [42,43]. Teraki et al. [23] reported an elevated serum level of IFN-γ only in patients with alopecia universalis in comparison with the controls. No significant difference in the serum level of IFN-γ was observed between patients with localized alopecia areata and the control group. In contrast to other studies, an analysis performed by Katagiri et al. [44] revealed a decreased level of IFN-γ mRNA in the peripheral blood mononuclear cells of patients with alopecia areata in comparison with healthy individuals.

Kasumagic-Halilovic et al. [39] reported a higher serum level of IFN-γ in patients with alopecia totalis/universalis compared to patients with localized alopecia areata. Ma et al. [37] reported an increased serum level of IFN-γ in patients with active alopecia areata in comparison with patients with stable alopecia areata and control subjects.

A significant decrease in the serum level of IFN-γ was observed in the group of patients with alopecia areata responding to DPCP therapy. However, no difference was observed in the serum level of IFN-γ before and after DPCP treatment in the non-responder group [34,36,38].

#### 2.1.3. Tumor Necrosis Factor (TNF)

TNF is an inflammatory cytokine produced by macrophages/monocytes and T and B lymphocytes during acute inflammation. It is responsible for a diverse range of signaling events within cells, leading to necrosis or apoptosis [45].

Numerous studies demonstrated an increased serum level of TNF in patients with alopecia areata compared to healthy controls [24,28,40,46,47,48,49]. Moreover, an increased expression of TNF mRNA was reported in peripheral blood mononuclear cells in patients with alopecia areata compared to healthy individuals [24,43].

A positive correlation between the serum level of TNF and disease severity was reported in the literature [40,47]. Indeed, a study performed by Alzolibani et al. [24] showed the serum level of TNF to be higher in patients with alopecia areata with the severity SALT score ≥ 25% compared to patients with the SALT score < 25%. Moreover, Rossi et al. [50] described a positive correlation between the expression of TNF level in peripheral blood mononuclear cells and the duration of the disease. According to Barahmani et al. [26] higher serum levels of TNF occurred in patients with alopecia areata and atopy compared to patients with alopecia areata without atopy.

#### 2.1.4. Interleukin 12 (IL-12)

IL-12 is a pro-inflammatory cytokine produced by dendritic cells, macrophages, and B cells in response to microbial pathogens [51]. It induces the production of IFN-γ by T and natural killer cells.

Some authors observed an increased serum level of IL-12 in patients with alopecia areata compared to healthy controls [26,34]. Moreover, others reported a higher expression of IL-12 mRNA in peripheral blood mononuclear cells in patients with alopecia areata compared to healthy controls [43].

Rossi et al. [50] demonstrated a positive correlation between IL-12 levels in peripheral blood mononuclear cells and the severity and duration of hair loss [52].

A study conducted by Gong et al. [34] revealed higher serum levels of IL-12 in responders compared to non-responders at baseline. A significant decrease in serum IL-12 level was detected in the responders after DPCP treatment, while in the non-responders the serum level of IL-12 increased.

#### 2.1.5. Interleukin 18 (IL-18)

IL-18 is a pleiotropic cytokine involved in the regulation of the innate and acquired immune response. It is produced by various hematopoietic and nonhematopoietic cells, including dendritic cells and macrophages. IL-18 is a potent inducer of IFN- γ in natural killer cells and CD4+ Th1 lymphocytes. It also modulates Th2 and Th17 cell responses, as well as the activity of CD8+ cytotoxic cells and neutrophils [53].

The majority of previously reported studies described no significant difference in the serum level of IL-18 between patients with alopecia areata and healthy controls [26,34,54]. However, Lee et al. [54] detected higher serum levels of IL-18 in patients with >50% of scalp hair loss compared to healthy controls and patients with ≤50% of scalp hair loss.

### 2.2. Th2 Cytokines

A summary of current literature considering the serum Th2 cytokine levels in patients with alopecia areata is presented in Table 2.

#### 2.2.1. Interleukin 4 (IL-4)

IL-4 is a multifunctional pleiotropic type I cytokine secreted by activated Th2 cells, basophils, eosinophils and mast cells. It plays a role in the regulation of T cell activation, differentiation, and proliferation and the survival of different T cell types [55].

An increased serum level of IL-4 was described in patients with alopecia areata in comparison with the control group in numerous of previously published studies [23,27,38,52]. In a study by Teraki et al. [23], higher serum levels of IL-4 were only detected in patients with localized alopecia areata in comparison with healthy controls. No significant difference in the serum level of IL-4 between patients with alopecia universalis and control subjects was reported. Moreover, Gautam et al. [25] reported a lower serum level of IL-4 in patients with alopecia areata compared to healthy subjects. A decreased expression of IL-4 mRNA was also observed in the peripheral blood mononuclear cells of patients with alopecia areata compared to healthy individuals [42,44].

According to Askin et al. [27], a decreased serum level of IL-4 was observed in patients with alopecia areata in comparison with baseline after tofacitinib therapy. Gong et al. [34] reported an increased serum level of IL-4 in the non-responder group when compared to the responders and healthy controls at baseline. After DPCP, an increase in the serum level of IL-4 was present in the responders in comparison with baseline, while in the non-responders no significant difference was detected. Conversely, Manimaran et al. [38] reported no significant difference in the serum level of IL-4 between the responders and non-responders at baseline. After DPCP, an increased serum level of IL-4 was present in the responders compared to baseline, while, in the non-responders, the serum level of IL-was was lower than at baseline.

Based on previous observations, the role of the serum IL-4 in patients with alopecia areata remains ambiguous; thus, further studies are needed to evaluate the role of this cytokine in pathogenesis of the disease.

#### 2.2.2. Interleukin 5 (IL-5)

IL-5 is a cytokine that is produced as a dimer and secreted by multiple cells, such as Th2 cells, mast cells, type 2 innate lymphoid cells, and eosinophils [56]. It plays a key role in the differentiation, development, and survival of eosinophils [56].

Studies conducted by Gong et al. [34,36] demonstrated a lower serum level of IL-5 in patients with alopecia areata in comparison with healthy controls. However, the serum level of IL-5 significantly increased in both, responders and non-responders, after DPCP therapy.

There are limited data considering the serum level of IL-5 in patients with alopecia areata; thus, further studies are needed to confirm these preliminary observations.

#### 2.2.3. Interleukin 6 (IL-6)

IL-6 is a pleiotropic cytokine involved in chronic inflammation and autoantibody production. It is produced by stromal cells, monocytes, and lymphocytes. The expression of IL-6 is increased by IL-1β and TNF, the stimulation of Toll-like receptors, and additional stress response proteins [57].

Numerous studies showed an increased serum level of IL-6 in patients with alopecia areata compared to healthy controls [26,31,35,47,48,58]. 

Tomaszewska et al. [35] observed a positive correlation between the serum level of IL-6 and the duration of alopecia areata.

#### 2.2.4. Interleukin 9 (IL-9)

IL-9 is a pleiotropic cytokine produced by a wide variety of cells including mast cells, natural killer cells, Th2, Th9, Th17, T regulatory cells, and type 2 innate lymphoid cells. It plays the main role in the immune responses against parasites and in the pathogenesis of allergic diseases [59].

To date, increased concentrations of serum IL-9 in patients with alopecia areata compared to healthy controls have only been reported in one study [38].

In a study conducted by Manimaran et al. [38], the serum level of IL-9 decreased after DPCP therapy in the responder group, while in the non-responders it remained at a higher level than in the controls.

The role of the serum IL-9 in alopecia areata needs to be confirmed in further studies.

#### 2.2.5. Interleukin 10 (IL-10)

IL-10 is a pluripotent cytokine considered as the most important anti-inflammatory cytokine found in the human immune response [49,58]. It is produced by different cell types including B and T lymphocytes, macrophages, monocytes, dendritic cells, and mast cells [58].

In numerous of previously published studies, an increased serum level of IL-10 was described in patients with alopecia areata compared to healthy controls [25,26,41,46]. Ma et al. [37] reported a decreased serum level of IL-10 in patients with active alopecia areata in comparison with patients with stable alopecia areata and healthy controls.

Gong et al. [34] observed no difference in the serum level of IL-10 between patients with alopecia areata and healthy controls at baseline. However, after DPCP treatment, an increased serum level of IL-10 was found in the responders compared to pre-treatment and controls. As regards the non-responders, no significant difference was present.

In numerous studies [22,34,49], serum level of IL-10 in patients with alopecia areata was comparable with healthy controls; thus, further studies are needed to confirm the role of this cytokine in alopecia areata.

#### 2.2.6. Interleukin 13 (IL-13)

IL-13 is a cytokine belonging to the alpha-helix protein family that is mainly produced by activated Th2 cells, mast cells, and basophils [60]. It was shown to upregulate MHC class II expression, promote IgE class switching, and inhibit inflammatory cytokine production. IL-13 shares multiple biological activities with IL-4 [61].

Some authors described an increased serum level of IL-13 in patients with alopecia areata in comparison with the control group [22,32,38]. Conversely, Loh et al. [28] reported lower serum levels of IL-13 in patients with alopecia areata than in control subjects.

Gong et al. [36] demonstrated no significant difference in the serum level of IL-13 between patients with alopecia areata and healthy controls at baseline. However, an increased pre-treatment serum level of IL-4 was observed in the non-responder group when compared to the responders. After DPCP therapy, the serum level of IL-13 increased in the responder group. In the non-responders, no difference in the serum level of IL-13 was detected before and after DPCP therapy. In a study by Manimaran et al. [38], a decrease in the serum level of IL-13 was observed in both, responders and non-responders, compared to baseline.

In a few studies [26,34], serum level of IL-13 in patients with alopecia areata was comparable with healthy controls; thus, further studies are needed to confirm the role of this cytokine in alopecia areata.

#### 2.2.7. Interleukin 17E (IL-17E)

IL-17E (IL-25) is member of the IL-17 cytokine family produced by innate cells and keratinocytes. It promotes the generation of Th2 cells and is involved in allergic inflammation.

Bain et al. [49] detected an increased serum level of IL-17E in patients with alopecia areata compared to healthy controls.

A positive correlation was observed between IL-17E level and hair loss severity and disease duration as well as depression score [49].

#### 2.2.8. Interleukin 31 (IL-31)

IL-31, is a member of the IL-6 family of cytokines, produced mainly by activated CD4+ T cells, activated Th2 cells in particular [49,62]. It induces proinflammatory cytokines and regulates cell proliferation [63].

Only one study performed by Bain et al. [49], described an increased serum level of IL-31 in patients with alopecia areata compared to healthy individuals.

#### 2.2.9. Interleukin 33 (IL-33)

IL-33 is an IL-1-like cytokine that plays an important role in Th2-associated immune responses [64].

The study performed by Bain et al. [49] demonstrated an increased serum level of IL-33 in patients with alopecia areata compared to healthy individuals.

### 2.3. Th17 Cytokines

A summary of current literature considering the serum Th17 cytokine levels in patients with alopecia areata is presented in Table 3.

#### 2.3.1. Interleukin 17 (IL-17)

IL-17, also known as IL-17A, is a pro-inflammatory cytokine secreted by CD4+ Th17 and CD8+ T cytotoxic cells [49]. It induces the production of granulocyte colony-stimulating factor (G-CSF) and chemokines, such as C-X-C motif chemokine ligands 1 and 2 [65].

Numerous studies showed an increased serum level of IL-17 in patients with alopecia areata compared to healthy controls [22,24,25,28,31,38,47,49,66,67,68]. Moreover, increased IL-17 gene expression was reported in the peripheral blood mononuclear cells of patients with alopecia areata compared to healthy controls [24].

A positive correlation was described between the serum level of IL-17 and disease severity in patients with alopecia areata [25,28,48]. El-Morsy et al. [66] reported a negative correlation between serum IL-17 and the age of the patients. Moreover, the serum level of IL-17 was decreased in patients with the current episode of hair loss longer than 2 years or with concomitant thyroiditis [31].

Morsy et al. [69] detected no difference in the serum level of IL-17 between patients with alopecia areata and healthy controls. However, a significant decrease in the serum level of IL-17 was reported in patients with alopecia areata after narrow-band ultraviolet B phototherapy. No correlation between the SALT score and IL-17 level was observed at baseline. However, a negative correlation was found between the SALT score and IL-17 level after phototherapy. Manimaran et al. [38] reported a significant decrease in the serum level of IL-17 after DPCP therapy in both responders and non-responders [38], while Askin et al. [27] described a decrease in the serum level of IL-17 after tofacitinib therapy when compared to baseline.

#### 2.3.2. Interleukin 17F (IL-17F)

IL-17F is a pro-inflammatory cytokine produced by a group of T helper 17 cells in response to their stimulation with IL-23 [70].

Only one study, by Bain et al. [49], reported an increased serum level of IL-17F in patients with alopecia areata compared to healthy controls.

#### 2.3.3. Interleukin 21 (IL-21)

IL-21 is a pleiotropic-type Th17 cytokine mainly produced by T cells and natural killer T cells. It exerts an effect on a broad range of cell types including CD4+ and CD8+ T cells, B cells, macrophages, monocytes, and dendritic cells [71].

Studies conducted by Bain et al. [49] and Atwa et al. [47] demonstrated an increased serum level of IL-21 in patients with alopecia areata compared to healthy controls.

#### 2.3.4. Interleukin 22 (IL-22)

IL-22 is a member of the IL-10 family of cytokines produced by Th17 cells, γδ T cells, natural killer cells, and innate lymphoid cells [47,72].

In a majority of previously published studies the serum level of IL-22 in patients with alopecia areata was comparable with healthy controls [28,34]. Only Atwa et al. [47] observed an increased serum level of IL-22 in patients with alopecia areata compared to healthy controls.

A positive correlation was described between the serum level of IL-22 and the duration of alopecia areata as well as depression [47,49].

Further studies are needed to confirm the role of the serum IL-22 in alopecia areata.

#### 2.3.5. Interleukin 23 (IL-23)

IL-23 is a member of the IL-12 family that induces the differentiation of naive CD4+ T cells into Th17 cells [73].

An increased serum level of IL-23 was reported in patients with alopecia areata compared to healthy individuals [46,49]. However, Bilgic et al. [46] performed logistic regression analyses and found a negative relationship between IL-23 levels and the presence of alopecia areata.

There are limited data considering the serum level of IL-23 in patients with alopecia areata, thus further studies are needed to confirm these preliminary observations.

#### 2.3.6. Transforming Growth Factor Beta (TGF- β)

TGF-β is a pleiotropic cytokine characterized by regulatory and inflammatory activity [74]. It induces Foxp3-positive regulatory T cells in the presence of IL-2, while in the presence of IL-6, it induces pathogenic IL-17 producing Th17 cells [50].

An increased serum level of TGF-β was observed in patients with alopecia areata compared to healthy controls [28,38]. However, a study conducted by Alzolibani et al. [24] showed that the serum level of TGF-β and TGF-β gene expression in peripheral blood mononuclear cells were lower in patients with alopecia areata when compared to healthy controls. Moreover, Tembhre et al. [22] reported a decreased serum level of TGF-β in patients with active alopecia areata compared to healthy controls.

According to Manimaran et al. [38], a decrease in the serum level of TGF-β was detected in the responders after DPCP therapy. As regards the non-responder group, no significant difference in the pre- and post-treatment cytokine level was reported.

Due to inconsistent data regarding the serum level of TGF-β in patients with alopecia areata, further studies are needed the evaluate the role of this cytokine in alopecia areata.

## 3. Discussion

It was reported that the local dysregulation of Th1, Th2 and Th17 cytokine secretion played an important role in the pathogenesis of alopecia areata [8,19]. Nevertheless, the assessment of the local cytokine profile requires an invasive procedure, such as scalp biopsy, so its role in clinical practice may be limited.

Recently, there has been a growing interest concerning systemic cytokines in patients with alopecia areata. To date, abnormal serum levels of numerous Th1, Th2, and Th17 cytokines have been reported in the literature [34,36,38]. However, the results of the previously publishes studies are not always consistent. To date, genome-wide association studies identified only a few susceptibility loci for alopecia areata associated with Th cytokines such as IL-2/IL-21 [18] and IL-13 [19].

**Figure 1:**
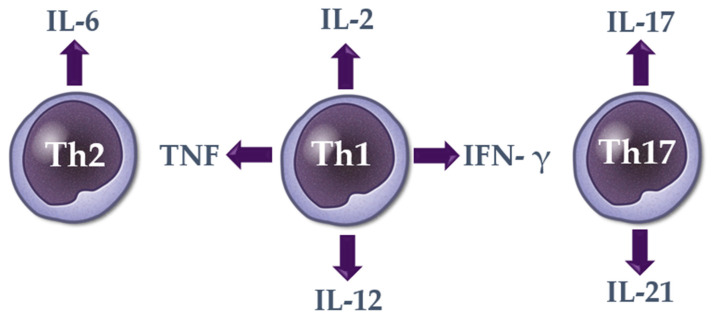
Systemic cytokines in alopecia areata. The role of the serum IL-18, IL-4, IL-5, IL-9, IL-10, IL-13, IL-17E, IL-17F, IL-21, IL-22, IL-23, IL-31, IL-33, and TGF-β has been indicated. Graphic by J. Taczała, MSc.

Based on our analysis, it may be suggested that alopecia areata is characterized by the dysregulation of systemic Th1 (IL-2, IFN-γ, TNF and IL-12), Th2 (IL-6), and Th17 (IL-17, IL-21) cytokines (Figure 1). The role of the serum IL-18, IL-4, IL-5, IL-9, IL-10, IL-13, IL-17E, IL-17F, IL-21, IL-22, IL-23, IL-31, IL-33, and TGF-β needs to be confirmed in further studies. An association between systemic cytokine dysregulation and the presence of alopecia areata was also confirmed by the fact that after the effective treatment of hair loss the serum level of various cytokines was restored and was similar to that in healthy controls [34,36,38].

The dysregulation of the serum level of Th1, Th2 and Th17 cytokines including proinflammatory markers, such as IL-2 and TNF, indicates that alopecia areata is a systemic inflammatory disorder not limited to the hair follicles. Indeed, a higher frequency of numerous consequences of systemic inflammation (such as metabolic syndrome, cardiovascular diseases, and depression) was reported in patients with alopecia areata compared to healthy controls [21,75].

According to the literature, a positive correlation was reported between the severity of alopecia areata and the serum level of various cytokines including IL-2 [25], TNF [40], IL-12 [50], IL-17 [25,28,48], and IL-17E [49] (Table 4).

Moreover, the serum level of numerous cytokines, such as IL-2 [22], IL-6 [35], TNF [50], IL-12 [50], IL-17E [49], and IL-22 [47] was shown to be associated with the duration of the disease (Table 5). Based on these observations, it may be suggested that the severity as well as the duration of hair loss has a direct impact on the severity of systemic inflammation in patients with alopecia areata. Therefore, patients with extensive forms of hair loss (such as alopecia totalis and universalis) or long-lasting disease are at a higher risk of developing the consequences of systemic inflammation.

The role of selected proinflammatory cytokines in the pathogenesis of alopecia areata may also be presumed on the basis of changes in their concentrations following treatment. In the study of Askin et al. [27], a significant decrease in the serum level of IL-2, IL-4, and IL-15 in patients with alopecia areata after tofacitinib was described. Manimaran et al. [27,38] reported a significant decrease in the serum level of IFN-γ, IL-17A, IL-9, TGF-β, and IL-13 after DPCP therapy in responders, while IL-4 significantly increased. Among the non-responders, only IL-17A and IL-13 levels were reduced. In the study conducted by Gong et al. [34], an increased serum level of IL-12 and decreased serum level of IL-10 after DPCP in non-responders were observed. Post-treatment DPCP responders exhibited significantly decreased IFN-γ and IL-12, and increased IL-4 and IL-10.

Numerous cytokine-targeting therapeutic modalities have been described as effective in alopecia areata such as JAK inhibitors, phosphodiesterase-4 inhibitor (apremilast), anti-IL-12/23 monoclonal antibody (ustekinumab), and anti-IL-17 monoclonal antibody (secukinumab) [8]. The efficacy of anti-IL-4 receptor alpha monoclonal antibody (dupilumab), anti-IL-13 monoclonal antibody (tralokinumab), and anti-IL-6 monoclonal antibody (siltuximab) in alopecia areata is still under investigation [8]. It may be hypothesized that evaluation of the serum level of various cytokines may be helpful in better selection of patients who will response to these therapeutic options. 

The prognostic role of systemic cytokine levels in patients with alopecia areata was also evaluated in the literature. To date, it has been shown that increased pre-treatment serum levels of IL-4 and IL-13 may be regarded as unfavorable predictors of a DPCP therapeutic effect [34,36]. Conversely, an increased serum level of IL-12 may be considered as a positive prognostic marker of DPCP therapy [34].

Further studies are needed to assess the cytokine profile in patients with alopecia areata and examine their role in the pathogenesis, clinical manifestations, and prognosis of the disease. Wider knowledge concerning the role of cytokines in alopecia areata will be helpful in introducing more specific therapeutic management.

## 4. Conclusions

Alopecia areata is characterized by systemic dysregulation of Th1 (IL-2, IFN-γ, TNF and IL-12), Th2 (IL-6), and Th17 (IL-17, IL-21) cytokines. The role of the serum IL-18, IL-4, IL-5, IL-9, IL-10, IL-13, IL-17E, IL-17F, IL-21, IL-22, IL-23, IL-31, IL-33, and TGF-β needs to be confirmed in further studies. Available data indicate the most significant role of serum IL-2, TNF, IL-12, IL-17, and IL-17E as markers of disease activity. The serum levels of IL-4, IL-12 and IL-13 may be useful as potential predictors of DPCP efficacy.

## Figures and Tables

**Table 1:** Summary of current literature considering the serum Th1 cytokine levels in patients with alopecia areata.

Cytokine	An Increased Serum Level (Number of Patients with Alopecia Areata)	A Decreased Serum Level (Number of Patients with Alopecia Areata)	Comparable to Healthy Controls (Number of Patients with Alopecia Areata)
IL-2	Teraki et al., 1996 (14), Barahmani et al., 2010 (269), Tembhre et al., 2013 (51) Alzolibani et al., 2016 (25), Kasumagić-Halilovic et al., 2018 (60), Gautam et al., 2020 (40), Aşkın et al., 2021 (61)	Tabara et al., 2019 (42)	Gong et al., 2020 (33)
IFN-γ	Omar et al., 2021 (72), Teraki et al., 1996 (14), Arca et al., 2004 (40), Barahmani et al., 2010 (269), Kasumagic-Halilovic et al., 2010 (60), Tembhre et al., 2013 (51), Ma et al., 2017 (100), Song et al., 2018 (30), Tabara et al., 2019 (42), Gong et al., 2020 (33), Manimaran et al., 2020 (33), Tomaszewska et al., 2020 (30), Gong et al., 2021 (33)	-	Loh et al., 2018 (55), Bain et al., 2020 (39)
TNF	Omar et al., 2021 (72), Alzolibani et al., 2016 (25), Atwa et al., 2016 (47), Bilgic et al., 2016 (40), Kasumagic-Halilovic et al., 2011 (60), Loh et al., 2018 (55), Bain et al., 2020 (39)	-	Barahmani et al., 2010 (269)
IL-12	Barahmani et al., 2010 (269), Gong et al., 2020 (33)	-	-
IL-18	Lee et al., 2010 (21)	-	Chodorowska et al., 2007 (14), Barahmani et al., 2010 (269), Gong et al., 2020 (33)

**Table 2:** Summary of current literature considering the serum Th2 cytokine levels in patients with alopecia areata.

Cytokine	An Increased Serum Level (Number of Patients with Alopecia Areata)	A Decreased Serum Level (Number of Patients with Alopecia Areata)	Comparable to Healthy Controls (Number of Patients with Alopecia Areata)
IL-4	Teraki et al., 1996 (14), Attia et al., 2010 (54), Manimaran et al., 2020 (33), Aşkın et al., 2021 (61)	Gautam et al., 2020 (40)	Barahmani et al., 2010 (269), Alzolibani et al., 2016 (25), Gong et al., 2020 (33)
IL-5	-	Gong et al., 2020 (33), Gong et al., 2021 (33)	Alzolibani et al., 2016 (25)
IL-6	Barahmani et al., 2010 (269), Atwa et al., 2016 (47), Bilgic et al., 2016 (40), Tabara et al., 2019 (42), Bain et al., 2020 (39), Tomaszewska et al., 2020 (30)	-	Ataseven et al., 2011 (43)
IL-9	Manimaran et al., 2020 (33)	-	Barahmani et al., 2010 (269)
IL-10	Cho et al., 2006 (21), Barahmani et al., 2010 (269), Bain et al., 2020 (39), Gautam et al., 2020 (40)	Ma et al., 2017 (100)	Ataseven et al., 2011 (43), Tembhre et al., 2013 (51), Gong et al., 2020 (33)
IL-13	Tembhre et al., 2013 (51), Song et al., 2018 (30), Manimaran et al., 2020 (33)	Loh et al., 2018 (55)	Barahmani et al., 2010, Gong et al., 2020
IL-17E (IL-25)	Bain et al., 2020 (39)	-	-
IL-31	Bain et al., 2020 (39)	-	-
IL-33	Bain et al., 2020 (39)	-	-

**Table 3:** Summary of current literature considering the serum Th17 cytokine levels in patients with alopecia areata.

Cytokine	An Increased Serum Level (Number of Patients with Alopecia Areata)	A Decreased Serum Level (Number of Patients with Alopecia Areata)	Comparable to Healthy Controls (Number of Patients with Alopecia Areata)
IL-17	Tembhre et al., 2013 (51), Alzolibani et al., 2016 (25), Atwa et al., 2016 (47) El-Morsy et al., 2016 (39), Elela et al., 2016 (40), Loh et al., 2018 (55), Tabara et al., 2019 (42), Bain et al., 2020 (39), Gautam et al., 2020 (40), Hatif et al., 2020 (58), Manimaran et al., 2020 (33)	-	Morsy et al., 2018 (20), Gong et al., 2020 (33), Aşkın et al., 2021 (61)
IL-17F	Bain et al., 2020 (39)	-	-
IL-21	Atwa et al., 2016 (47), Bain et al., 2020 (39)	-	-
IL-22	Atwa et al., 2016 (47)	-	Loh et al., 2018 (55), Gong et al., 2020 (33)
IL-23	Bilgic et al., 2016 (40), Bain et al., 2020 (39)	-	Loh et al., 2018 (55), Gautam et al., 2020 (40)
TGF-β	Loh et al., 2018 (55), Manimaran et al., 2020 (33)	Tembhre et al., 2013 (51), Alzolibani et al., 2016 (25)	-

**Table 4:** The serum cytokines associated with severity of alopecia areata.

The serum cytokines associated with severity of alopecia areata
IL-2 [25]
TNF [40,47]
IL-12 [50]
IL-17 [25,28,48]
IL-17E [49]

**Table 5:** The serum cytokines associated with severity of alopecia areata.

The serum cytokines associated with duration of alopecia areata
IL-2 [22]
IL-6 [35]
TNF [50]
IL-12 [50]
IL-17E [49]
IL-22 [47]
